# Aerosol-generating otolaryngology procedures and the need for enhanced PPE during the COVID-19 pandemic: a literature review

**DOI:** 10.1186/s40463-020-00424-7

**Published:** 2020-05-11

**Authors:** Paul Mick, Russell Murphy

**Affiliations:** 1 Department of Surgery, University of Saskatchewan, 2708-103 Hospital Drive, Saskatoon, SK S7N 0W8 Canada; 2 Department of Surgery, University of Saskatchewan, Wall Street ENT Clinic, 230-140 Wall Street, Saskatoon, SK S7K 1N4 Canada

**Keywords:** COVID-19, PPE, Personal protective equipment, AGMP, Aerosol generating medical procedure, Otolaryngology-head and neck surgery, Otolaryngology, ENT

## Abstract

**Background:**

Adequate personal protective equipment is needed to reduce the rate of transmission of COVID-19 to health care workers. Otolaryngology groups are recommending a higher level of personal protective equipment for aerosol-generating procedures than public health agencies. The objective of the review was to provide evidence that a.) demonstrates which otolaryngology procedures are aerosol-generating, and that b.) clarifies whether the higher level of PPE advocated by otolaryngology groups is justified.

**Main body:**

Health care workers in China who performed tracheotomy during the SARS-CoV-1 epidemic had 4.15 times greater odds of contracting the virus than controls who did not perform tracheotomy (95% CI 2.75–7.54). No other studies provide direct epidemiological evidence of increased aerosolized transmission of viruses during otolaryngology procedures. Experimental evidence has shown that electrocautery, advanced energy devices, open suctioning, and drilling can create aerosolized biological particles. The viral load of COVID-19 is highest in the upper aerodigestive tract, increasing the likelihood that aerosols generated during procedures of the upper aerodigestive tract of infected patients would carry viral material. Cough and normal breathing create aerosols which may increase the risk of transmission during outpatient procedures. A significant proportion of individuals infected with COVID-19 may not have symptoms, raising the likelihood of transmission of the disease to inadequately protected health care workers from patients who do not have probable or confirmed infection. Powered air purifying respirators, if used properly, provide a greater level of filtration than N95 masks and thus may reduce the risk of transmission.

**Conclusion:**

Direct and indirect evidence suggests that a large number of otolaryngology-head and neck surgery procedures are aerosol generating. Otolaryngologists are likely at high risk of contracting COVID-19 during aerosol generating procedures because they are likely exposed to high viral loads in patients infected with the virus. Based on the precautionary principle, even though the evidence is not definitive, adopting enhanced personal protective equipment protocols is reasonable based on the evidence. Further research is needed to clarify the risk associated with performing various procedures during the COVID-19 pandemic, and the degree to which various personal protective equipment reduces the risk.

## Background

During the coronavirus disease 2019 (COVID-19) pandemic, personal protective equipment (PPE) worn by health care workers is critical for reducing transmission of the infection in health care settings, particularly when aerosol-generating medical procedures (AGMP) are being performed. An aerosol is a suspension of fine solid particles or liquid droplets in air or another gas. Within an aerosol, viral droplet nuclei can travel long distances and remain in the air for long periods of time. Aerosols are not as effectively filtered by surgical masks, and can be breathed directly into the lungs. For transmission to occur, it is not enough for viral material to exist in droplet nuclei; the virus must remain viable. Whether or not COVID-19 remains viable in aerosols (and for how long) is still being investigated, but the balance of evidence indicates that *betacoronaviradae* such as the 2003 SARS coronavirus (SARS-CoV-1) are viable in aerosols [1]. Many otolaryngology procedures are thought to be aerosol-generating [2]. When healthcare workers are at risk of transmission of infection from aerosols, “airborne” (rather than droplet) precautions are required [3].

Otolaryngologists who are susceptible to being infected with COVID-19 and who are working in close proximity to infected tissues for lengthy periods may be exposed to large infectious doses. COVID-19 infects the upper aerodigestive tract with the highest viral loads occurring in the nasal cavities [4]. The surgeon’s nose, throat, and conjunctiva (all potential routes of transmission) [1, 5] are typically within 30–60 cm of the patient’s upper respiratory mucosa. During AGMP, as a surgeon gets closer to the source of the aerosol, particle density increases exponentially according to principles of diffusion [6].

The association between infectious dose and disease severity has not yet been determined. Analogous novel viral respiratory viruses, however, may provide a degree of evidence. The basic reproductive numbers (the expected number of cases directly generated by one individual in a population where all individuals are susceptible) for SARS-CoV-1 and COVID-19 appear to be similar and thus comparisons are reasonable [7, 8]. In animal studies, increasing the initial exposure to SARS-CoV-1 increased the risk that mice developed the infection [9]. Greater initial exposures to SARS-CoV-1 [10], MERS coronavirus [11] and influenza [12] resulted in more severe disease. In at least one recent study, a higher concentration of COVID-19 in the nasal passages (i.e., higher viral load) was associated with increased risk of more severe disease and death [13]. Viral load, however, is measured after the onset of infection and thus is not a proxy for infective dose.

During the pandemic, health care agencies such as the World Health Organization, U.S. Centers for Disease Control and the Public Health Agency of Canada [3, 14, 15] are responsible for defining AGMP and rationing PPE when demand is greater than supply. The lists of AGMP often do not specifically include otolaryngology procedures. National otolaryngology organizations and other ENT groups [16] have published otolaryngology-specific AGMP lists and PPE guidelines that call for a greater levels of protection than the public health agencies. For example, Givi et al and the Canadian Society of Otolaryngology-Head and Neck Surgery [2] call for airborne precautions when performing AGMP on patients for whom the index of suspicion for COVID-19 infection is not high, whereas the World Health Organization, the U.S. Centers for Disease Control, and the Public Health Agency of Canada do not [3, 14, 15]. Givi et al also suggest that health care workers use powered air purifying respirators (PAPRs) when available for AGMP performed on patients with probable or confirmed COVID-19, in contrast to public health agencies that are either silent on the issue or suggest PAPRs are not needed [17].

We are members of the Division of Otolaryngology in Saskatoon, Saskatchewan. We were invited by the local health authority to provide evidence that a.) demonstrates which otolaryngology procedures are aerosol-generating, and that b.) clarifies whether the higher level of PPE advocated by otolaryngology groups is justified. The following serves as a summary of our submission.

## Main text

### Part 1: aerosol-generating otolaryngology procedures

#### Is COVID-19 transmitted via aerosols?

Respiratory aerosols typically consist of droplet nuclei less than 5 μm in size [18]. Droplets fall to the ground at rates inversely proportional to their size. A 10 μm diameter particle settles in 8.2 min, compared to 1.5 h for a 3 μm diameter particle, and 12 h for a 1 μm particle [19]. Thus, unless rooms are well ventilated, aerosolized droplets can become more concentrated over time. For an infection to be transmitted via aerosol, the organism must be able to survive within the droplet nuclei until it is deposited onto the mucous membrane of a susceptible individual either via inhalation or direct contact.

The World Health Organization has cautioned that more studies are needed to confirm if COVID-19 is transmitted via aerosols [20], however an April 1, 2020 report from the U.S. National Academies of Science, Engineering and Medicine suggests it is likely [21]. The letter cites studies in which COVID-19 RNA was detected in air samples in hospital rooms of patients with COVID-19 [22]. A widely cited experimental study indicates that COVID-19 can remain viable in aerosols for hours [5], but has been criticized since the methods used to aerosol the virus in the experiment are not reflective of AGMP or natural cough [20]. A case report of a trans-nasal pituitary adenoma excision performed in China before widespread introduction of strict PPE provides anecdotal evidence of aerosolized transmission of COVID-19. During the case, fourteen Chinese health care workers were reportedly infected by the patient (who was mildly symptomatic pre-operatively), who was later confirmed to have COVID-19. Transmission occurred to workers who were both inside and outside the operating room [23]. During the SARS-CoV-1 epidemic, the largest nosocomial outbreak in Hong Kong occurred with a clear spatial pattern of infection that matched ventilatory patterns of the hospital floor, suggesting aerosolized transmission was likely [24]. A similar study showed that the pattern of spread of a large community outbreak of SARS-CoV-1 matched the ventilatory pathways from the apartment of the index case [25].

#### Aerosolized transmission of COVID-19 during surgeries and endoscopy of the upper aerodigestive tract

Research about AGMP has arisen from and been motivated by the need to protect health care workers during previous pandemics. Cohort and case-control studies comparing the rates of transmission from patients to health care workers who perform certain procedures versus health care workers who do not provide direct evidence of the risk conferred by the procedures. Experiments demonstrating that various procedures generate aerosols provide more limited evidence since they do not prove that transmission occurs via the airborne route.

After the AIDS epidemic of the 1980s there was concern regarding the transmission of blood-borne viral illnesses during surgery. Experiments showed that electrocautery, bone drilling, ultrasonically activated (Harmonic) devices, and suction irrigation create aerosolized blood droplets and tissue particles [26–29]. There is no epidemiological evidence, however, that the human immunodeficiency virus can be transmitted via aerosolized blood droplet nuclei [30]. Experiments have also shown that intranasal and temporal bone drilling aerosolizes bone, blood and mucosa [26, 28, 31]. Workman et al applied fluorescein inside the nasal cavity of cadaveric specimens, performed various surgical procedures, and measured aerosol spread outside of the nostrils using a blue-light filter and digital image processing. Intranasal drilling but not cold instrumentation or microdebriding produced fluorescein aerosols that could be detected up to 60 cm from the nostrils [31]. During temporal bone drilling the spread of particles might be greater since the walls of the nasal cavity likely prevent the spread of some material. It is not known if the respiratory mucosa lining the middle ear and mastoid air cell system is involved in COVID-19, but because the rest of the airway is involved, it appears likely that the lining of the eustachian tube, middle ear, and mastoid air cell system are also contaminated [32, 33]. .For these reasons, the use of use of high speed drills during mastoidectomy should be considered an AGMP during COVID-19.

During the SARS-CoV-1 epidemic, it was initially thought that transmission occurred primarily via contact or large respiratory droplets. It was observed, however, that transmission to health care workers occurred despite the use of contact and droplet precautions, particularly during procedures suspected to be aerosol-generating such as endotracheal intubation [34, 35]. A meta-analysis of observational studies evaluating the risk of transmission of SARS-CoV-1 during the epidemic showed that health care workers performing endotracheal intubation, non-invasive ventilation, tracheotomy and manual ventilation before intubation were significantly more likely than health care workers not involved in these procedures to contract the disease [36]. Only one case-control study of front-line health care workers caring for SARS-CoV-1 patients in China contributed to the “meta-analysis” of tracheotomy [37]. In the univariate analysis, 6/85 cases (who had IgG against SARS-CoV-1) versus 11/646 controls (who did not have IgG against SARS-CoV-1) had performed tracheotomies during the epidemic (Odds ratio 4.15, 95% CI 2.75, 7.54).

The odds ratio for bronchoscopy, on the other hand, did not reach significance (pooled OR 1.3, 95% CI 0.5, 14.2). Many public health agencies and professional organizations [38], however, list bronchoscopy as an aerosol generating procedure. The World Health Organization appears to classify bronchoscopy [39] as an AGMP based on a study comparing the rate of tuberculin skin test conversion among pulmonology and infectious diseases fellows graduating in 1983 during a resurgence of tuberculosis in the United States. Seven of 62 (11%) pulmonology fellows versus one of 42 (2.4%) infectious diseases fellows reported having converted tuberculin skin tests during their fellowships [40]. It was not clear that the pulmonology fellows were infected as a result of performing bronchoscopies. A 2009 study during the H1N1 influenza outbreak measured the amount of viral RNA in the air in the vicinity of H1N1 positive patients undergoing bronchoscopy and other procedures, compared to controls. The concentration of viral RNA was not significantly increased during bronchoscopy or any other procedure studied. The authors wrote that their study may have been underpowered to detect small differences in aerosol concentrations [41].

If bronchoscopy is aerosol-generating, it may be due to the suctioning usually involved with the procedure. Air currents moving across the surface of a film of liquid generate droplets at the air-liquid interface, with the size of the droplets inversely proportional to the velocity of the air [39]. It is for this reason that any procedure that involve open suctioning of the airway is usually classified as aerosol-generating.

There do not appear to be any studies that directly assess whether diagnostic nasopharyngoscopy produces aerosols in patients infected with respiratory viruses, and/or if it is associated with increased risk of airborne transmission of respiratory viruses to healthcare workers. Workman et al performed an experiment in which they pushed an atomizer device from the cranium of a cadaver through the cribriform plate and into the nasal cavity, plunged the syringe “at maximal pressure” to inject aerosolized fluorescein into the nasal cavity, then performed intra-nasal endoscopy and measured the spread of fluorescein out the nostrils. Various masks that were modified to allow passage of the endoscope were placed on the cadaver head in front of the nostrils. It is not known whether their methods accurately mimic the situation in patients with COVID-19. They did find, however, that the masks reduced the spread of fluorescein outside the nostris [31]. Despite the lack of evidence, in the COVID-19 era diagnostic endoscopy of the upper airways is often listed as an AGMP by health care agencies, likely because of its perceived similarities to bronchoscopy and because the endoscope travels through tissues with high COVID-19 viral loads [2, 42]. In contrast to bronchoscopy, however, many endoscopic procedures of the upper aerodigestive tract do not require suctioning. Further evidence is needed to understand the degree to which endoscopy of the upper aerodigestive tract generates aerosols.

#### Generation of aerosols during cough, pursed lip breathing and normal breathing: implications for outpatient procedures

Most ENT outpatient procedures induce coughing due to deep instrumentation and/or excessive mucous or blood that triggers the cough reflex. The jet of droplets and aerosols expelled by a cough can hit nearby health care workers at high volume and velocity, and at close range. The frequency of cough is higher in a patient infected with COVID-19, since it is a symptom of the infection [43]. The World Health Organization considers cough to be aerosol-generating [44], a position that is supported by a number of studies [45–50]. The average distribution of droplet sizes expelled during cough ranges on average between 0.58–5.42 μm, with multi-modal peaks at 1, 2 and 8 μm. Larger droplets may partially evaporate during the jet expulsion from the mouth to produce smaller droplet nuclei [45]. Aerosols are also generated by “pursed lip” breathing methods, often adopted by patients who have epistaxis to avoid aspirating blood trickling posteriorly and into the throat [51].

Aerosols can be produced by normal breathing as air passes over respiratory mucosa [52–54], through the reopening of closed small airways to form small airborne droplets [55], and/or through fluid film rupture in the bronchioles [56]. During normal breathing, the lungs filter out most larger droplets from being exhaled [53]. As might be expected, coughing produces more aerosolized droplets than normal breathing or talking [53]. Breathing rate and age are both positively correlated with breath aerosol concentration, but do not completely explain the variability observed between individuals [56].

Head and neck physical examinations and the collection of nasopharyngeal swab samples are not typically classified as AGMP [17]. The fact that aerosols are produced during normal breathing combined with the close proximity required to perform these procedures do, however, provide support for recommendations from otolaryngology groups that airborne precautions should be taken by health care workers performing head and neck examinations in patients who have suspected or known COVID-19 [16].

### Part 2: evidence clarifying if enhanced PPE are needed for otolaryngology AGMP

Givi et al and the Canadian Society of Otolaryngology-Head and Neck Surgery suggest adhering to airborne precautions when performing AGMP on patients whose COVID-19 status is unknown or who have low risk of infection during the pandemic [2, 16]. They also recommend PAPRs (if available) to perform AGMP on patients with probable or confirmed COVID-19 [2, 16]. The World Health Organization [57], CDC [14] and Public Health Agency of Canada [15] do not make these recommendations.

Occupational health professionals are often tasked with determining the type of PPE needed in novel circumstances arising in various industries. The CDC through the National Institute for Occupational Safety and Health (NIOSH) [58] and the Canadian Center for Occupational Safety and Health [59] recommend “control banding” as a qualitative or semi-qualitative technique used to guide the implementation of workplace control measures. In control banding assessments, the potential for harm is determined by 1.) the consequences of exposure; 2.) the concentration of toxin; and 3.) the risk of exposure. Operations that expose workers to a greater potential for harm demand more stringent control measures. The consequences of COVID-19 infection to individuals are well described elsewhere [43] but range from mild illness to death. If health care workers become sick they can pass the infection to others, propagating the pandemic, and are no longer available to assist on the front lines. The increased risk of exposure to high concentrations of aerosols during otolaryngology AGMP has already been discussed. Thus, the following section focuses on the third element, the risk of exposure to COVID-19, and the likelihood that the different PPE recommended by the different groups alters the risk.

#### The risk of exposure to COVID-19 when a patient’s COVID-19 status is unknown

A significant proportion of individuals infected with COVID-19 are either pre-symptomatic (they have not developed symptoms yet) or asymptomatic (they never develop symptoms). The mean incubation of COVID-19 period is 5–6 days, with a range of 1–14 days [43]. A well-known study of 3063 passengers on the quarantined Diamond Princess cruise ship showed that 52% of 634 persons who tested positive for COVID-19 had no symptoms at the time of testing [60]. On March 31, 2020, the director of the U.S. Centers for Disease Control (CDC) stated that the percentage of people in the general population who have COVID-19 but do not have symptoms is 25% [61]. This estimate ranges from 12.6% in China [62] to 50% in Iceland, where a very high proportion of the population (5%) has been tested for COVID-19 and thus the results may be more reflective of reality [63].

Pre-symptomatic carriers can transmit disease. On April 1, the CDC reported the results of an investigation of all 243 cases of COVID-19 reported in Singapore between January 23 and March 16. Seven clusters of cases were identified in which pre-symptomatic transmission was the most likely cause of secondary cases [64]. It is estimated that 44% of transmission could occur before the first symptoms [65].

The true number of cases of COVID-19 in the population is unknown but is assuredly much higher than the number of cases confirmed by testing and reported to government agencies due to limitations in population sampling and test sensitivity [66]. It is therefore likely that a significant proportion of patients presenting to the health care system for various reasons but who do not complain of symptoms of COVID-19 will be infected with the virus and can transmit it to health care workers for many months to come.

The sensitivity and specificity of commonly performed COVID-19 diagnostic tests has not been definitively determined in part because a safe “gold standard” comparator has yet to be developed. Variability in sampling due to technical difficulties swabbing the nasopharynx or because of changes in the viral load throughout the course of illness may affect the sensitivity of the test. A negative result thus does not necessarily rule out infection. If the test is positive, it is likely correct, although it is possible that though cross-contamination from other patients or lab workers could result in false positive results [66]. The positive- and negative-predictive values of the test depend in part on the local true prevalence of COVID-19.

For the reasons stated above, recommendations for airborne precautions for AGMP performed on patients whose COVID-19 status is unknown during the pandemic appear to be reasonable according to the precautionary principle [67]. It is not clear when such precautions should be rescinded. Published epidemiological projections suggest that similar to previous pandemics, even after the current wave of new cases subsides, outbreaks will recur throughout the world over at least the next year until herd immunity and/or an effective vaccination program is established [68].

#### The risk of exposure of COVID-19 using powered air-purifying respirators, reusable elastomeric respirators and filtering facepiece respirators (N95 masks)

Powered air-purifying respirators (PAPRs), reusable elastomeric respirators and filtering facepiece respirators (e.g., N95 masks) represent different methods of filtering out aerosols in the air. A PAPR, which costs about USD 1400, contains a battery-powered high-efficiency particulate air filter that delivers clean air into a hood or a full face mask, and blows off exhaled air. The hood is either hard and tight-fitting or loose. The risk of leakage with PAPRs is negligible and, unlike reusable elastomeric respirators and N95 masks, there is no need for a fit test or additional eye protection since the head is completely enclosed within the system [69]. This feature of the PAPR benefits individuals who fail fit tests and those whose religious beliefs prevent them from shaving. Decontamination protocols for PAPRs must be in place and adhered to meticulously before they are re-used [69]. Resuable elastomeric respirators, which typically cost <USD 100, are used more commonly in heavy industry than health care. Such devices are made to meet National Institute for Occupational Safety and Health (NIOSH) standards, and are defined by the ability of the device to filter out oil or non-oil particulate. They may either cover the lower half of the face (and require additional eye protection) or cover the entire face. Respirators are classified according to their particulate filtration efficiency; Both reusable elastomeric respirators and filtering facepiece respirators come in P100 (99.97% filtration efficiency) and N95 (95% filtration efficiency) varieties [70]. The familiar N95 mask costs USD 1.50 and can thus be more widely distributed during a pandemic. No studies exist that directly compare the different respirators’ abilities to prevent transmission of viral illness in the health care setting. Such studies would need to incorporate doffing and reprocessing procedures in the experimental design, since there is high risk of transmission if doffing is performed incorrectly, and the risk may be modified by the type of PPE used [71]. .Experimental studies in the occupational health literature have been performed that compare how well different respirators filter aerosols (typically sodium chloride aerosols) in simulated industrial environments. The protection factor is a measure of the ratio of airborne contaminant inside and outside the respirator. The U.S. Occupational Health and Safety Administration reports that the assigned protection factor (i.e., the workplace level of respiratory protection that a respirator is expected to provide to employees in the context of a “continuing, effective respiratory protection program”) for PAPRs ranges between 25 and 10,000, versus 10 for filtering facepiece respirators, 10 for half mask elastomeric respirators, and 50 for full facepiece elastomeric respirators [72]. Other reports similarly demonstrate the superior performance of PAPRs or elastomeric respirators in simulated workplace environments compared to N95 filtering facepiece respirators [73, 74].

A major risk with filtering facepiece respirators and reusable elastomeric respirators is that the air tight seal can leak during a procedure, compromising their performance. In a study of 8 subjects each tested on six different N95 masks, thermal imaging showed that leakage (mainly in the nasal and malar region) occurred in all failed leak tests [13] and the majority (26 of 35) passed fit tests [75]. The risk of leakage would be expected to increase with the length of time the N95 mask is worn, for example during long otolaryngology cases.

There is concern that the unfiltered exhaust from PAPRs may increase the risk of transmission of virus particles to patients from users who are unknowingly infected with COVID-19, but this risk would likely be diminished if PAPR users wore surgical masks [76]. PAPRs may be cumbersome to use, may fog up, and prevent the use of headlights, but these concerns are also true of masks, face shields and goggles.

The balance of evidence suggests that PAPRs and full facepiece elastomeric respirators, when properly used, doffed and reprocessed, would be expected to reduce the risk of transmission of infection and/or severity of illness by reducing exposure to COVID-19 during high risk otolaryngology cases, compared to N95 masks. Cost-effectiveness studies comparing different respirator types have not been performed but it may be feasible for hospitals to acquire a limited number of suits for use during high risk procedures. A 2010–2012 survey across six American states that included 98 hospitals indicated that 85% of the hospitals had PAPRs available [77]. The prevalence of PAPRs in Canadian hospitals is unknown. As initial outbreaks of COVID-19 diminish there is an opportunity for hospitals to acquire PAPRs and develop protocols for their use in preparation for future COVID-19 cases and outbreaks.

#### The reuse of disposable N95 masks

Given the shortages of disposable N95 filtering facepiece respirators worldwide, there has been emphasis on their reuse. For example, Givi et al state that it may be appropriate to reuse N95 masks after AGMPs performed on patients at low risk of having COVID-19 [16]. Experimental models show that virus survival on N95 masks depends on time elapsed and relative humidity [78]. SARS-CoV-1 can survive for up to 28 days on medical equipment in low temperature and low humidity environments [79]. There has been no published research on experiments involving COVID-19. A variety of decontamination methods have been proposed, from using heat and steam, hydrogen peroxide vapor, UV light, and letting the mask sit for days before repeat usage [80–82]. The use of ultraviolet light (UV-C) has been proposed owing to its ability to penetrate the materials found in N95 masks [83]. Using a surrogate virus, Fisher and Shaffer described that a minimum dose of 1000 j m^− 2^ of UV-C is required to cause a 3 log reduction in a surrogate virus [84]. Vaporized hydrogen peroxide has also been suggested as method useful for decontaminating materials, and has been demonstrated to reduce infectious titers of mammalian viruses to less than ten 50% tissue culture infective doses (TCID_50_) [85]. The CDC has provided statement that decontamination with either UVC, vaporized hydrogen peroxide, or moist heat could be considered by health care institutions in an emergency [86].

## Conclusion

Of the myriad of different procedures performed by otolaryngologists, only tracheotomy has specifically been found to pose an increased risk of airborne viral transmission to health care workers, in a study examining exposure to AGMP performed on patients with SARS-CoV-1 [36]. A large amount of indirect evidence, however, demonstrates that most surgical procedures of the upper aerodigestive tract create aerosolized biological particles through the use of suction, cautery, and other powered instrumentation. In patients infected with COVID-19, aerosolized particles would likely contain viral material because of high viral loads in the upper aerodigestive tract. Cough, pursed lip- and natural breathing also produce aerosols. The close proximity of a physician performing outpatient ENT procedures to an infected patient’s mouth and nose adds further hazard. Emerging evidence suggests that COVID-19 is viable in aerosolized droplet nuclei and that a larger infective dose (which may result from prolonged exposures during long procedures) may increase the severity of disease.

At any given time, a significant proportion of patients infected with COVID-19 will not have symptoms. Negative COVID-19 test results may not be accurate. Travel history will no longer be an important predictor of disease as border restrictions severely reduce the frequency of travel. It is thus reasonable, based on the precautionary principle, for health care workers performing AGMP during the pandemic to observe airborne precautions regardless of whether a patient is known to have COVID-19 or not.

Otolaryngology groups have advocated for the use of respirators with greater filtration capabilities than N95 masks, when available. Experimental studies conclude that PAPRs and reusable elastomeric respirators have greater filtration capabilities when used, doffed and reprocessed properly. Studies have not been performed that compare rates of transmission and cost-effectiveness between different types of respirators in the real-world circumstance of a pandemic. PAPRs are significantly more expensive than other types of respirators but the investment appears to be reasonable for lengthy high risk procedures.

Local institutional recommendations and region-specific epidemic characteristics must be taken into account when determining PPE use. Otolaryngology-Head and Neck Surgery is a relatively small specialty and institutional pandemic planning committees may be unaware of the level of PPE required for otolaryngology procedures. Otolaryngologists should participate in or communicate with PPE planning committees so that their viewpoints are reflected in institutional protocols. Regional epidemic characteristics (e.g., the prevalence of cases and trend of the epidemic curve) should also be considered when determining the level of PPE required, especially when a patient’s COVID-19 status is unknown. Patients who do not have a diagnosis of COVID-19 are obviously more likely to be asymptomatic or pre-symptomatic carriers if they live in regions where the prevalence is high. The reported prevalence, however, may underestimate the true value especially in jurisdictions where the rate of screening testing is low. Furthermore, even if the epidemic curve shows signs of flattening or diminishing, recurrent outbreaks may occur suddenly and without warning, especially if physical distancing and/or other public health measures are not being effectively followed [87]. The most cautious approach, if possible, would therefore be to adhere to a higher standard of PPE until the pandemic is over.

There is an opportunity to perform research to quantify the risk of COVID-19 transmission from patients to health care workers performing aerosol-generating otolaryngology procedures during the COVID-19 pandemic. The most important studies would be cohort studies that aim to estimate the risk of developing COVID-19 from performing otolaryngology AGMPs, and whether different types of PPE, training and/or doffing procedures modify the outcomes. Measuring the level of aerosolized viral particles in rooms where AGMPs are being performed on patients with COVID-19 would provide indirect evidence of the degree to which these procedures put health care workers at risk of aerosolized transmission, and whether exposure concentration affects risk of infection and/or severity of disease.

## Data Availability

Not applicable.

## Abbreviations

PPE: Personal protective equipment
COVID-19: Coronavirus disease 2019
SARS-CoV-1: Severe acute respiratory syndrome coronavirus-1
PAPR: Powered air-purifying respirator
CDC: Centers for Disease Control and Prevention
CSOHNS: Canadian Society of Otolaryngology-Head and Neck Surgery
